# MicroRNA‐503 promotes angiotensin II‐induced cardiac fibrosis by targeting Apelin‐13

**DOI:** 10.1111/jcmm.12754

**Published:** 2016-01-12

**Authors:** Yuhong Zhou, Lin Deng, Dandan Zhao, Lanlan Chen, Zhen Yao, Xiaowei Guo, Xue Liu, Lifang Lv, Bing Leng, Wei Xu, Guofen Qiao, Hongli Shan

**Affiliations:** ^1^ Department of Pharmacology (The State‐Province Key Laboratories of Biomedicine‐Pharmaceutics of China, Key Laboratory of Cardiovascular Research, Ministry of Education)Harbin Medical UniversityHarbinChina; ^2^ Department of CardiologyThe Second Affiliated Hospital of Harbin Medical UniversityHarbin Medical UniversityHarbinChina

**Keywords:** Apelin‐13, miR‐503, cardiac fibrosis

## Abstract

Cardiac fibrosis is a major cause of heart failure. MicroRNAs (miRs) are important epigenetic regulators of cardiac function and cardiovascular diseases, including cardiac fibrosis. This study aimed to explore the role of miR‐503 and its mechanisms in regulating cardiac fibrosis. miR‐503 was found up‐regulated in the mouse LV tissues subjected to transverse aortic constriction (TAC) and in neonatal cardiac fibroblasts (CFs) cultured with Angiotension II. The role of miR‐503 in regulating CF cell proliferation and/or collagen production in mice neonatal CFs were determined using an MTT assay and RT‐PCR respectively. Forced expression of miR‐503 increased the cellular proliferation and collagen production in mice neonatal CFs. The effects were abrogated by cotransfection with AMO‐503 (a specific inhibitor of miR‐503). Injection of antagomiR‐503 elevated cardiac function and inhibited the expression of connective tissue growth factor (CTGF) and transforming growth factor (TGF)‐β in the TAC mice. Additional analysis revealed that Apelin‐13 is a direct target of miR‐503, as the overexpression of miR‐503 decreased the protein and mRNA expression levels of Apelin‐13. In the CFs with pre‐treatment of AngII, we transfected AMO‐503 into the cells treated with siRNA‐APLN. siRNA‐APLN abolished the effects of AMO‐503 on the production of collagen I and III and the expression of TGF‐β and CTGF. Furthermore, pre‐treatment of CFs with Apelin‐13 (1–100 nmol/l) inhibited angiotensin II‐mediated collagen production and activation of CTGF and TGF‐β. So we conclude that miR‐503 promotes cardiac fibrosis *via* miR‐503‐Apelin‐13‐TGF‐β‐CTGF‐collagen production pathway. Thus, miR‐503 is a promising therapeutic target for reducing cardiac fibrosis.

## Introduction

Cardiac fibrosis is mainly induced by various cardiac injuries, such as myocardial infarction, chronical hypertension and so on. During the process, normal cardiac residential fibroblasts (CFs) and/or circulating fibroblasts become myofibroblast cells. These myofibroblasts produce and secret excess amounts of extracellular matrix (ECM) commonly including collagen I, collagen III and fibronectins, *etc*. 1. Cardiac fibrosis can alter many structural and functional aspects of the heart, which accompanies with myocardial remodelling and leads to eventual heart failure 2, 3. Therefore, great efforts have been made to better understand this pathological process and to uncover its underlying molecular mechanisms.

MicroRNAs (miRNAs) are a class of small non‐coding RNAs binding to the complementary sequences in the 3′‐untranslated regions of target mRNAs of protein‐coding genes to repress protein expression at the post‐transcription level. More and more evidences have been shown that miRNAs have critical roles in participating and regulating various cardiovascular diseases, such as cardiac arrhythmia 4, cardiac hypertrophy 5, 6, cardiac injuries 7 and finally the heart failure 8. It has been reported that several miRNAs are involved in the development and progression of cardiac fibrosis. miR‐21 and miR‐29 provoke the development of fibrosis, while miR‐133 and miR‐101 9, 10, 11, 12 suppress the fibrotic responses of CFs *via* the regulation of various fibrosis‐related proteins. Many miRNAs have been found involved specifically in cardiac fibrosis. As Dr. Dai mentioned that an improved understanding of the role of multiple miRs targeting several different signalling pathways will provide novel and exciting therapeutic modalities 13.

Apelin is a secreted peptide belonging to the adipokine family. It regulates cardiovascular functions *via* its binding to the APJ receptor 14. Apelin has many various fragments and Apelin‐13 is the main one. It has been reported that Apelin‐13 plays a central role in CF activation. Apelin‐13 inhibits transforming growth factor (TGF)‐β‐induced phenotypic switching of fibroblast‐to‐myofibroblast *via* a SphK1‐dependent mechanism 15. Apelin‐13 increases angiogenesis and attenuates cardiac fibrosis and hypertrophy, and also improves cardiac repair post‐MI by up‐regulating SDF‐1α/CXCR‐4 in vascular progenitor cells 16. However, little is known about the regulatory factors that modulate Apelin‐13, or the mechanisms underlying this process. To study potential epigenetic regulation of Apelin‐13 protein level, we performed Target Scan and found miR‐503 is match candidate.

miR‐503 was first identified in human retinoblastoma tissues 17. Considering the seed sequence similarities and the genomic organization, it is possible to classify miR‐503 as part of the extended miR‐16 family 18. Members of this family are miR‐15a/b, miR‐16, miR‐195, miR‐424 and miR‐497. The expression of miR‐503 was increased in human parathyroid carcinomas and adenocortical carcinomas 19, 20. It is critical for the differentiation of myoblasts and myogenesis. *In vivo*, miR‐503 is up‐regulated in myocardial ECs from diabetic GK rats and in ECs resident in ischaemic limb muscles of diabetic mice 21, 22. It is also up‐regulated in ischaemic limb skeletal muscles and the plasma of diabetic patients with end‐stage critical limb ischaemia 21. It was believed that miR‐503 was involved in the regulating cellular differentiation and modulating the cell cycle.

Based on the information provided by Target Scan, we suggested that miR‐503 was a major miR that controls the expression of Apelin‐13 in CFs. To explore this hypothesis, by using transfection and real‐time quantitative RT‐PCR analysis, we tested whether miR‐503 can alter the expression level of Apelin‐13 in neonatal CFs. With a series of biochemical analyses, we demonstrated that miR‐503 acts directly on Apelin‐13 mRNA and this miR‐503‐dependent down‐regulation of Apelin‐13 can lead to the repression of the TGF‐β‐connective tissue growth factor (CTGF) pathway, which ultimately induced excessive ECM deposition and cardiac fibrosis. This study implies that manipulating the expression level of miR‐503 represents a promising therapeutic strategy potentially for the treatment of cardiac fibrosis.

## Materials and methods

### Ethics statement

All animal experiments were approved by the Committee on Animal Experimentation of Harbin Medical University (Approval ID: DEC6121) and were conducted in accordance with international guidelines regarding animal experimentation.

### Animals and housing conditions

Male C57BL6/J mice aged 8–10 weeks and weighing 20–25 g were obtained from the Animal Centre of Harbin Medical University. During the experiment, the animals were kept on a 12 hr light: 12 hr dark cycle and were granted *ad libitum* access to food and water.

### Surgical procedures of TAC

To induce pressure‐overload heart hypertrophy, animals were subjected to transverse aortic constriction (TAC). Adult Male C57BL6/J mice were anaesthetized with pentobarbital (65 mg/kg, intraperitoneal injection). Animals were placed in the supine position. After successful endotracheal intubation, the cannula was connected to a volume‐cycled rodent ventilator (UGO BASILE S.R.L. Italy). The chest was opened and the thoracic aorta was identified. A 7–0 silk suture was placed around the transverse aorta and tied around a 26‐gauge blunt needle which was subsequently removed. The chest was closed and the animals were kept ventilated until recovery of autonomic breath. All surgical procedures were performed under sterile conditions. The cholesterol‐conjugated miR‐503 antisense and negative control (antagomiR‐503 and antagomiR‐NC, respectively) were purchased from RiboBio (Guangzhou, China). After TAC surgery, we injected antagomiR‐503 (30 mg/kg bw in 50 μl) and antagomiR‐NC (30 mg/kg bw in 50 μl) one time a day by tail vein on days 1, 2, 8, 9, 15 and 16. After 28 days, we evaluate their cardiac function and measure the fibrotic factors using heart tissue. The heart was quickly excised and weighted in cold (4°C) buffer. The left ventricle tissue was then rapidly frozen in liquid nitrogen and stored at −80°C for subsequent western blot or real‐time PCR analysis. All procedures involving animals and their care were approved by the Institutional Animal Care and Use Committee of Harbin Medical University, China.

### Echocardiographic measurements

Four weeks after the injection of antagomiR‐503 and antagomiR‐NC, we used transthoracic echocardiography with an ultrasound machine (Vivid 7,GE Medical, Horten, Norway) equipped with a 10‐MHz phased‐array transducer to test the LV function. LV systolic diameter, left ventricular diastolic diameter (LVDd), interventricular septum diastolic thickness (IVSd) and interventricular septum systolic thickness (IVSs) were measured, and left ventricular ejection fraction (EF) and fractional shortening (FS) were calculated from M‐mode recording. After functional measurement, mice were killed with overdose sodium phenobarbital. The hearts were collected and put in liquid nitrogen or 4% paraformaldehyde for use.

### Cell culture

Mice neonatal CFs were isolated and cultured as previously described 11. Briefly, hearts from 2‐ to 3‐day‐old mice were finely minced and digested using type II collagenase (120 units/ml; Worthington Biochemical Corp., Lakewood, NJ, USA) for 10 min. every time, supernatant fluid were collected. Fibroblasts were isolated by removal of myocytes through selective adhesion of non‐myocytes at a 1.5 hrs pre‐plating interval. Cardiac fibroblasts were maintained in DMEM supplemented with penicillin and streptomycin (1%) and fetal bovine serum (FBS) (10%). Cardiac fibroblasts at the second passage were used in the experiments.

### Transfection

miR‐503 and their antisense oligonucleotides, AMO‐503 were synthesized by GenePharma (GenePharma Co. Ltd., Shanghai, China). A scrambled RNA was used as negative control. Transfections of miR‐503 and AMO‐503 were performed with Lipofectamine 2000 (Invitrogen, Life Technologies, Carlsbad, CA, USA) according to the procedure specification. The same procedures described elsewhere 12.

### Construction of the plasmid expressing small interfering RNA (siRNA) for rat Apelin

The siRNA for Rattus norvegicus APLN was constructed by using plasmid pGCsilencer^™^ U6/Neo/GFP/RNAi Expression Vector (GeneChem Co. Ltd., Shanghai, China), which contained a human U6 promoter, a GFP reporter gene and a neomycin resistance gene to enable antibiotic selection in mammalian cell. Three target sequences of rattus APLN mRNA were constructed. They were APLN RNAi‐: GGCUAGAAGAAGGCAACAUTT‐AUGUUGCCUUCUUCUA GCCTT. These different short‐hairpin RNA (shRNA) sequences were inserted into pGCsilencer^™^ U6/Neo/GFP/RNAi plasmid. To verify the sequence specificity of the pGCsilencer^™^ U6/Neo/GFP/RNAi‐specific shRNA system, a control‐shRNA vector (pGCsi.U6/neo/GFP RNAi‐NC Plasmid) was constructed by insertion of a sequence that expresses a shRNA with no significant homology to APLN mRNA gene and which had the sequence not present in the mouse, human or rat genome databases. The control insert sequence was: 5′‐TTCTCCGAACGTGTCACGTTTCAAGAGAACGTGACACGTTCGGAGAA‐3′.

### Luciferase assays

To construct reporter vectors bearing miRNA‐target sites, we first obtained fragments of the 3′UTRs of Apelin (rat and mouse) containing the exact target sites for miR‐503 by PCR amplification. Apelin 3′UTRs was inserted into the multiple cloning sites downstream the luciferase gene (HindIII and SacI sites) in the pMIR‐REPORT^TM^ luciferase miRNA expression reporter vector (Ambion, Inc., Austen, TX, USA.) to form chimeric plasmid.

After that, 1 μg of the chimeric plasmid (firefly luciferase vector), 0.1 μg PRL‐TK (TK‐driven Renilla luciferase expression vector) and the appropriate miRNAs or their inhibitors were cotransfected with lipofectamine 2000 (Invitrogen, Life Technologies, Carlsbad, CA, USA) into HEK‐293 cells (1 × 105/well). Luciferase activities were measured with a dual luciferase reporter assay kit (Promega, Madison, WI, USA) on a luminometer (GloMax^TM^ 20/20), 48 hrs following transfection. For all experiments, transfection took place 24 hrs after starvation of cells in serum‐free medium. The normalized luciferase activity relative to control group was used to demonstrate the alteration of mRNA transcription.

### Real‐time reverse transcript polymerase chain reaction

After the experimental treatment, total RNA from cultured fibroblasts and cardiac tissues were isolated using Trizol reagent (Invitrogen) 12. Total RNA (0.5 μg) was then reverse transcribed using High‐Capacity cDNA Reverse Transcription Kit (Applied Biosystems, Foster City, CA, USA) to obtain cDNA. The RNA levels of Apelin‐13, TGF‐β1, collagen I, collagen III and CTGF were determined using SYBR Green I incorporation method on ABI 7500 fast Real‐Time PCR system (Applied Biosystems), with β‐actin as an internal control. The forward and reverse PCR oligonucleotide primers are listed in Table 1. The level of miR‐503 were tested using TaqMan MicroRNA Assay Kit (Applied Biosystems), with U6 as an internal control.

**Table 1:** Primers Designed With Mice‐specific Genes for RT‐PCR

Name	Accession no.	Primer Sequence
Collagen I	HB1211290011	F: AAGAAGACATCCCTGAAGTCA R: TTGTGGCAGATACAGATCAAG
Collagen III	HB1211290011	F: TTGGGATGCAGCCACCTTG R: CGCAAAGGACAGATCCTGAG
Apelin‐13	HB1210100052	F: GGCTAGAAGAAGGCAACATGC R: CCGCTGTCTGCGAAAATTTCCT
APJ	HB1210100052	F: CCACCTGGTGAAGACTCTCTACA R: CTGACGTAACTGATGCAGGTG
CTGF	HG1403181051	F: AATCTCCACCCGAGTTACCA R: AACTTAGCCCTGTATGTCTTCAC
TGF‐β1	HG1403181051	F: GACTCTCCACCTGCAAGACC R: CCCTGTATTCCGTCTCCTTG
miR‐503	HB1209260003	RT: GTCGTATCCAGTGCGTGTCGTGGAGTCGGCAATTGCACTGGATACGACCTGCAGT F: GCGTAGCAGCGGGAACAGT R: CCAGTGCGTGTCGTGGAGT

### Western blot analysis

Western blotting was performed as previously described 11. Briefly, cells were lysed with modified RIPA buffer for 30 min. on ice, and cell lysates were subsequently centrifuged at 12,000 × g for 20 min. at 4°C. The supernatant was transferred to a fresh ice‐cold tube, and protein concentrations were determined *via* a Bio‐Rad protein assay (Bio‐Rad, Hercules, CA, USA). Equal concentrations of proteins were mixed with SDS sample buffer and denatured at 95°C for 5 min. Samples were resolved with 8% SDS‐page gel for protein. Gels were then transferred onto nitrocellulose membrane paper, and the membranes were blocked with 5% non‐fat dried milk in TTBS (0.1% Tween‐20) for 1 hr. Following blocking, the blots were incubated overnight with primary antibodies at 4°C. The anti‐Apelin‐13, anti‐CTGF and anti‐TGF‐β antibodies were obtained from Santa Cruz Biotech (Santa Cruz, CA, USA). Following washing with TTBS, the membranes were incubated with HRP‐conjugated secondary antibodies (1:5000; Santa Cruz Biotechnology) for 1 hr. Finally, the membranes were washed again with TTBS, and the blots were determined using an enhanced chemiluminescence detection system (ECL; GE Healthcare, Bio‐Science AB, Uppsala, Sweden).

### CFs proliferation assay

Both cell counting and a 3‐(4,5‐dimethylthiazol‐2‐yl)‐2,5‐diphenyltetrazolium bromide (MTT) cell vitality assay were used to measure CFs proliferation. CFs were seeded onto 96‐well tissue culture plates (5 × 10^3^ cells per well) in DMEM with 10% FBS. Following culturing in serum‐free Dulbecco‐modified Eagle medium for 24 hrs, Angiotensin II (AngII, 100 nmol/l; Sigma‐Aldrich, St. Louis, MO, USA), either alone or in combination with miR‐503 (50 nmol/l), miR‐503+AMO‐503 (100 nmol/l, the specific inhibitor of miR‐503). Following 48 hrs of incubation, CFs were trypsinized and counted with a haemocytometer. The CFs was then treated with MTT (5 mg/ml) for 4 hrs at 37°C. The amount of metabolized MTT was determined using a microplate reader.

### Statistical analysis

All values are expressed as the means ± S.E.M. Differences between the two groups were determined *via* a Student's *t*‐test. Comparisons between more than two groups, data were assessed using one‐way anova, followed by a Bonferroni correction. A value of *P* < 0.05 was considered statistically significant.

## Results

### Up‐regulation of miR‐503 in TAC hearts, Ang II and serum‐treated mice CFs

**Figure 1:**
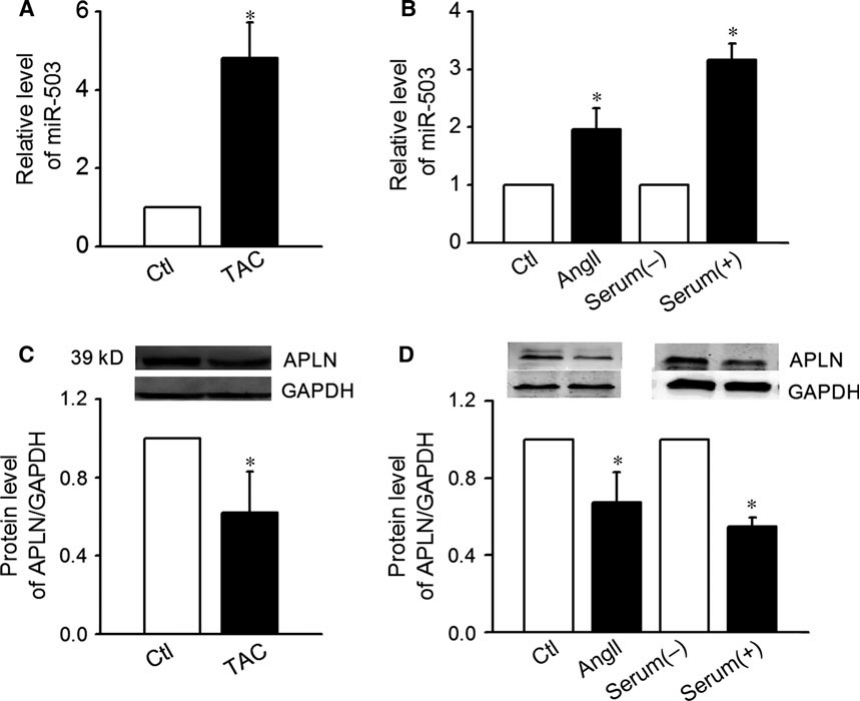
Expression levels of miR‐503. (**A**) The up‐regulated expression of miR‐503 in the hearts of mice that underwent transverse aortic constriction (*n* = 12, *P < 0.05 control (Ctl)). (**B**) The up‐regulated expression of miR‐503 in mice cardiac fibroblasts (CFs) treated with serum or Ang II (*n* = 8, *P < 0.05 *versus* serum (−), control (Ctl). (**C**) Protein expression levels of Apelin‐13 in the hearts of mice that underwent transverse aortic constriction (*n* = 12, *P < 0.05 *versus* Ctl). (**D**) Protein expression levels of Apelin‐13 in CFs treated with serum or AngII (*n* = 8, *P < 0.05 *versus* serum (−), control (Ctl).

The expression of miR‐503 was first detected in TAC cardiac ventricles and cultured CFs treated with serum and AngII. Twenty‐eight days following TAC, the expression of miR‐503 in cardiac ventricles was increased compared with sham‐operated animals (Fig. 1A).

miR‐503 expression was higher in cultured in the serum containing medium compared to no serum condition (Fig. 1B). Treatment with 100 nmol/l of AngII also increased the expression of miR‐503 (Fig. 1B). These findings suggested miR‐503′s possible involvement in CFs proliferation. Apelin‐13 protein expression was decreased in both TAC cardiac ventricles and CFs treated with Ang II and serum (Fig. 1C and D).

### miR‐503 promoted the proliferation of CFs

**Figure 2:**
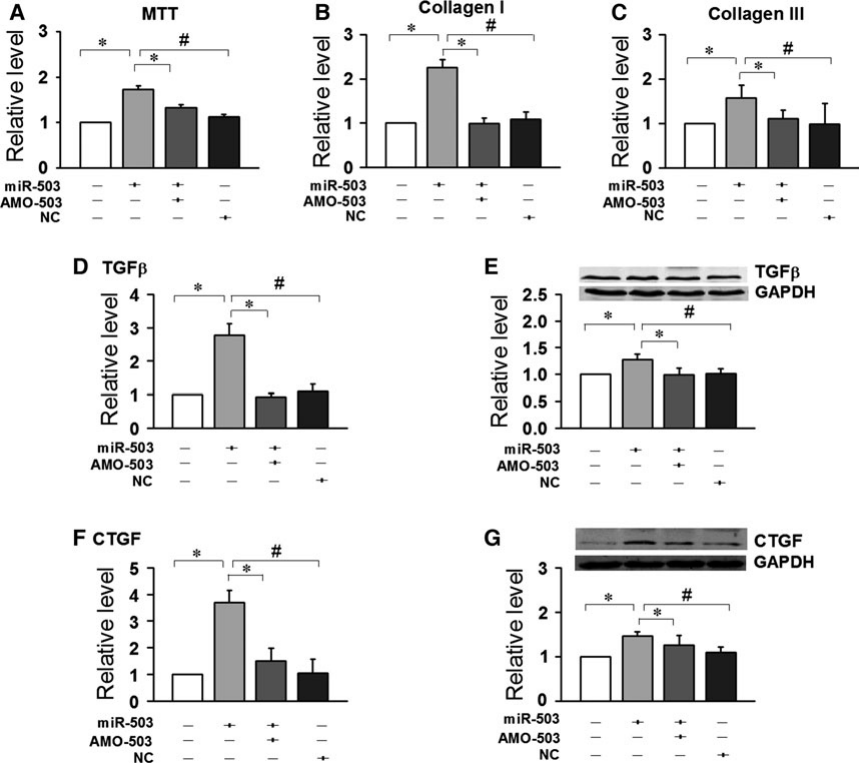
Fibrotic effects of miR‐503. The overexpression of miR‐503 promoted the proliferation of neonatal mice cardiac fibroblasts (CFs), as well as the expression of collagen I and collagen III in CFs. (**A**) MTT test. (**B** and **C**) mRNA levels of collagen I and III by quantitative reverse transcription‐polymerase chain reaction. The effects of miR‐503 on the protein and mRNA levels of transforming growth factor‐β (TGF‐β) (**D** and **E**) and its downstream molecule, CTGF (**F** and **G**). Data are expressed as the means ± S.E.M.; *n* = 5–6. NC indicates negative control. #*P* < 0.05 *versus* NC; *P < 0.05 *versus* miR‐503.

The effects of miR‐503 on neonatal CFs proliferation were evaluated *via* an MTT assay. Overexpression of miR‐503 (50 nmol/l) resulted in increased CFs proliferation (*n* = 32, *P* < 0.05), whereas cotransfection with AMO‐503 (100 nmol/l), a specific inhibitor of miR‐503, abrogated its effects (Fig. 2A).

Similarly, the overexpression of miR‐503 elevated the mRNA levels of collagen I and collagen III, while up‐regulated the expressions of CTGF and TGF‐β. Cotransfection with AMO‐503 suppressed collagen I, collagen III, CTGF and TGF‐β up‐regulation (Fig. 2B–G).

### Inhibition of antagomiR‐503 on TAC‐induced cardiac fibrosis

**Figure 3:**
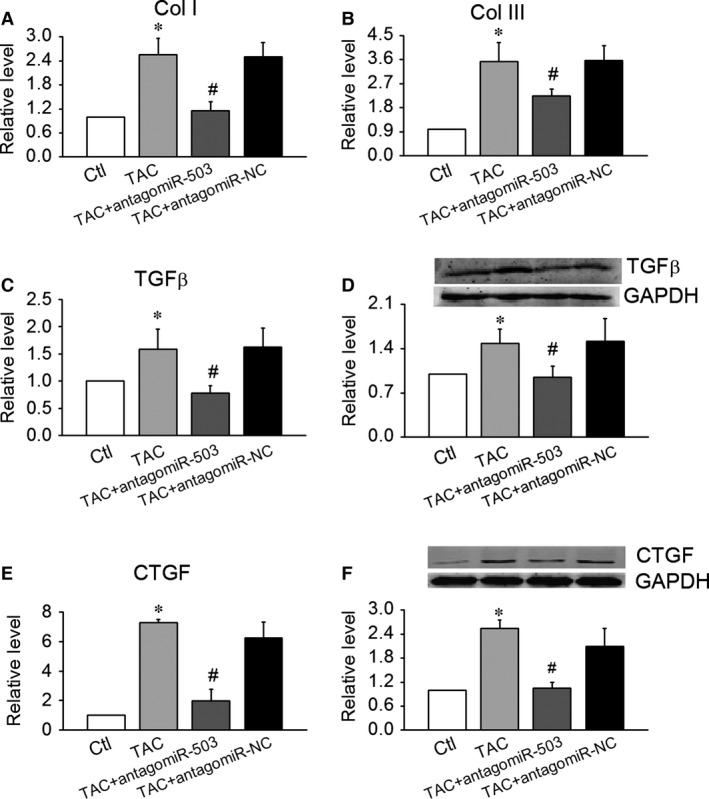
Inhibition of antagomiR‐503 on TAC‐induced cardiac fibrosis. (**A** and **B**) Effects of antagomiR‐503 on mRNA levels of collagen I and III in TAC mice. (**C**–**F**) Effects of antagomiR‐503 on the protein and mRNA levels of transforming growth factor‐β (TGF‐β) (**C** and **D**) and its downstream molecule, CTGF (**E** and **F**). Data are expressed as the means ± S.E.M.; *n* = 5–6. NC indicates negative control. *P < 0.05 *versus* control (Ctl); #*P* < 0.05 *versus* TAC.

To support the role of miR‐503 in heart fibrosis development, an *in vivo* experiment is performed to investigate whether administration of antagomiR‐503 (miR‐503 inhibitor) could decrease TAC‐induced cardiac fibrosis. After TAC surgery, we injected the antagomiR‐503 30 mg/kg one time a day by tail vein on days 1, 2, 8, 9, 15 and 16. Twenty‐eight days after surgery, we evaluate their cardiac function and measure the fibrotic factors using heart tissue. As Table 2 showed, EF and FS was elevated and LVDd and LVDs were decreased in the antagomiR‐503 group, compared to TAC group. The results showed that the antagomiR‐503 could improve the cardiac function induced by TAC. The Real‐time PCR and Western Blot results showed that the antagomiR‐503 inhibited the production of collagen I and III and down‐regulated the expression of TGF and CTGF, compared to TAC group (As shown in the Fig. 3A–F).

**Table 2:** Echocardiography of TAC mice after treatment with antagomiR‐503 for 4 weeks

	Sham (*n* = 6)	TAC (*n* = 6)	TAC+antagomiR‐503 (*n* = 6)	TAC+antagomiR‐NC (*n* = 6)
LVDd, mm	3.14 ± 0.12	5.34 ± 0.19^a^	3.76 ± 0.19^b^	4.94 ± 0.23
LVSd, mm	2.07 ± 0.12	3.26 ± 0.15^a^	2.68 ± 0.24^b^	3.06 ± 0.14
IVSd, mm	0.72 ± 0.02	0.75 ± 0.04	0.83 ± 0.02	0.68 ± 0.05
IVSs, mm	0.85 ± 0.03	0.91 ± 0.03	0.91 ± 0.03	0.89 ± 0.04
FS, %	44.1 ± 1.5	21.1 ± 2.72^a^	32.9 ± 3.46^b^	29.1 ± 2.72
EF, %	84.1 ± 3.7	42.9 ± 3.17^a^	66.1 ± 5.43^b^	54.6 ± 3.23

a *P <* 0.05 *versus* sham.

b *P <* 0.05 *versus* TAC.

Data are expressed as mean ± S.E.M.

LVDd: left ventricular diastolic diameter; LVSd: left ventricular systolic diameter; IVSd: interventricular septum diastolic thickness; IVSs: interventricular septum systolic thickness; FS: fractional shortening; EF: ejection fraction.

### The 3′UTR of Apelin‐13 mRNA was the target of miR‐503

**Figure 4:**
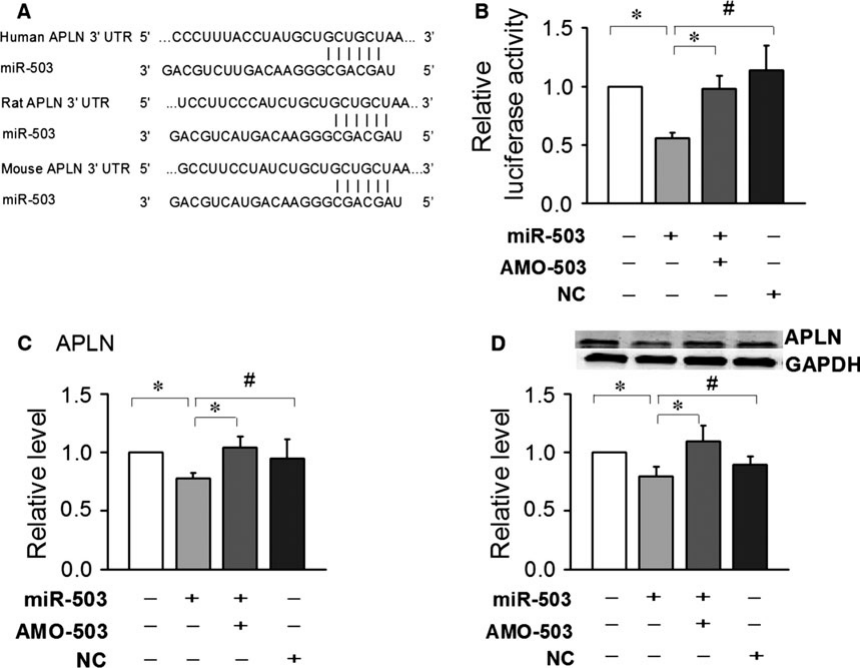
Experimental establishment of Apelin‐13 as a target of microRNA miR‐503. (**A**) Sequences of miR‐503 binding sites in Apelin‐13. (**B**) The luciferase reporter assay results depicting the activities of chimeric vectors carrying the luciferase gene and a fragment of the Apelin‐13 3′‐untranslated region from rats containing the binding sites of miR‐503. (**C**) is mRNA and (**D**) is the protein level of Apelin‐13 induced by overexpression of miR‐503 in cultured neonatal mice cardiac fibroblasts (CFs). Data are expressed as the means ± S.E.M.; *n* = 4. NC indicates negative control. #*P* < 0.05 *versus* NC; *P < 0.05 *versus* miR‐503.

Our computational analysis predicted that miR‐503 has the potential to suppress Apelin‐13: the 3′‐untranslated region of Apelin‐13 mRNA contains one binding site for miR‐503 among humans, rats and mice (Fig. 4A). In this study, we confirmed the regulation of Apelin‐13 by miR‐503 using a luciferase assay. We found that miR‐503 (50 nmol/l) significantly inhibited the activity of luciferase vectors containing the 3′‐untranslated region of Apelin‐13 mRNA, which was rescued by co‐application of AMO‐503 (100 nmol/l). The specificity action of miR‐503 was determined by the absence of changes, an absence that coincided with the use of NC‐a negative control (Fig. 4B).

The 3′UTR of Apelin‐13 mRNA was a direct target of miR‐503. We then determined if the expression of Apelin‐13 was affected by the overexpression of miR‐503. Our data indicated that mRNA levels were changed by transfected miR‐503 (Fig. 3C), whereas the protein levels of Apelin‐13 were decreased (Fig. 3D).

**Figure 5:**
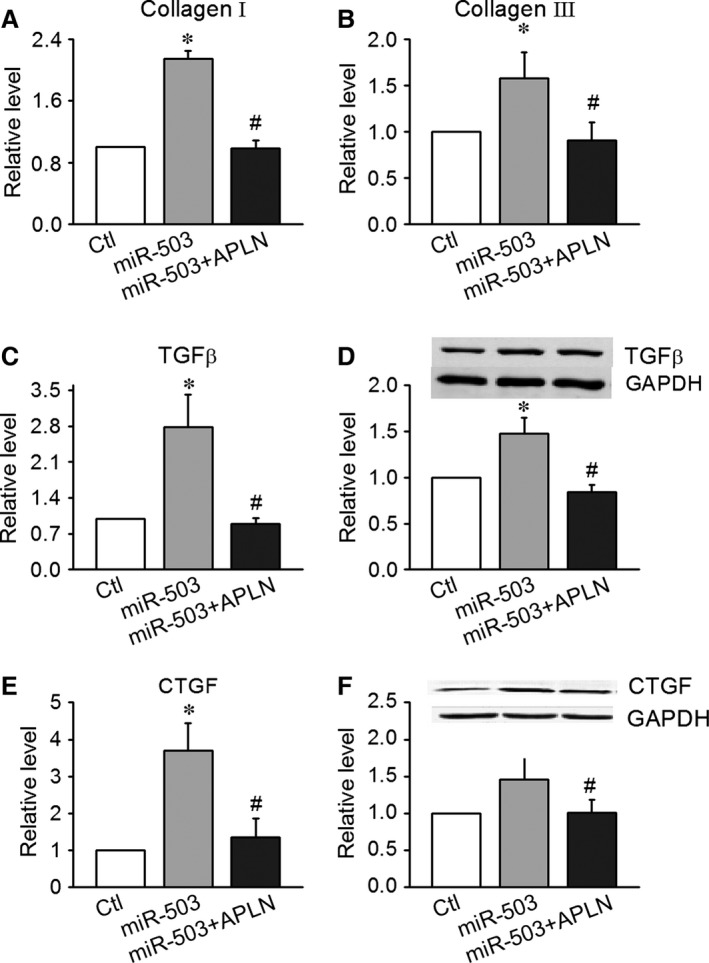
Involvement of Apelin‐13 in miR‐503‐mediated promotion of CF proliferation. (**A** and **B**) Apelin‐13 (100 nmol/l) decreased the expression of collagen I and collagen III in CFs transfected with miR‐503. (**C** and **D**) Apelin‐13 inhibited the expression of transforming growth factor‐β (TGF‐β) at the protein and mRNA levels in CFs transfected with miR‐503. (**E** and **F**) Apelin‐13 inhibited the expression of CTGF, a molecule downsteam of TGF‐β, at the protein and mRNA levels in CFs transfected with miR‐503. Data are expressed as the means ± S.E.M.; *n* = 5–6. *P < 0.05 *versus* control (no treatment); #*P* < 0.05 *versus* miR‐503.

To further confirm the role played by Apelin‐13 in the proliferative actions of miR‐503, we pre‐treated CFs with 100 nmol/l Apelin‐13 prior to the transfection of miR‐503 and examined any subsequent CFs proliferation. Transfection with miR‐503 (50 nmol/l) increased the production of collagen I and III and up‐regulated the protein and mRNA expression levels of TGF‐β and CTGF. Co‐application of Apelin‐13 100 nmol/l successfully suppressed collagen I and III, TGF‐β and CTGF (Fig. 5A–F). These data suggest that Apelin‐13 plays an important role in miR‐503‐mediated fibrosis.

### Involvement of Apelin‐13 in miR‐503‐mediated promotion of CFs proliferation

**Figure 6:**
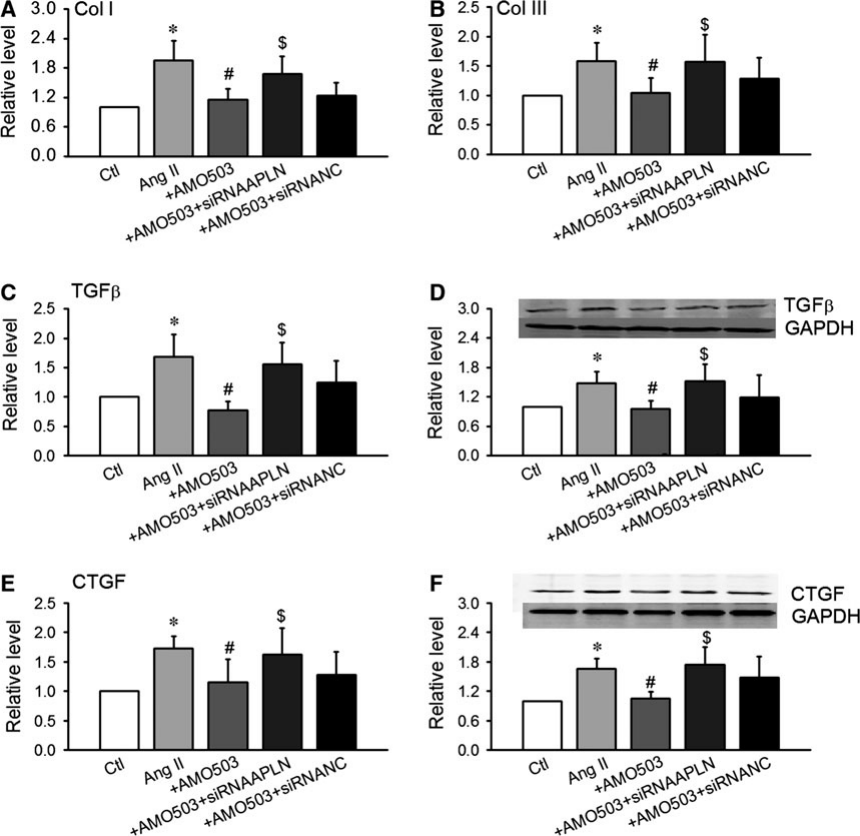
siRNA‐APLN abolished the effects of AMO‐503 in the CFs with pre‐treatment of Ang II (100 nmol/l). We transfected AMO‐503 (100 nmol/l) into the cells treated with siRNA‐APLN (50 nmol/l) and siRNA‐NC (50 nmol/l). (**A** and **B**) AMO‐503 inhibited the production of collagen I and III induced by Ang II in CFs. (**C**–**F**) Effects of antagomiR‐503 on the protein and mRNA levels of transforming growth factor‐β (TGF‐β) (**C** and **D**) and its downstream molecule, CTGF (**E** and **F**). Data are expressed as the means ± S.E.M.; *n* = 5–6. NC indicates negative control. *P < 0.05 *versus* control (Ctl); #*P* < 0.05 *versus* Ang II, $*P* < 0.05 *versus* Ang II AMO503.

To further prove miR‐503/APLN/TGF‐β‐CTGF pathway, we transfected AMO‐503 into CFs treated with siRNA‐APLN (APLN blocker) and detected the expression of TGF‐β and CTGF. In the CFs with pre‐treatment of Ang II (100 nmol/l), we transfected AMO‐503 (100 nmol/l) into the cells treated with siRNA‐APLN (50 nmol/l) and siRNA‐NC (50 nmol/l) respectively. AMO‐503 inhibited the production of collagen I and III and the expression of TGF‐β and CTGF induced by Ang II. Cotransfection with siRNA‐APLN abolished the effects of AMO‐503 on the production of collagen I and III and the expression of TGF‐β and CTGF. Cotransfection with siRNA‐NC did not affect the production of collagen I and III and the expression of TGF‐β and CTGF in the CFs with treatment of AMO‐503 (Fig.6A‐F). These studies *in vitro* proved the role of miR‐503 in heart fibrosis development by APLN/TGF‐β and CTGF pathway.

### Apelin‐13 inhibits angiotensin II‐induced fibrotic responses via a TGF?‐CTGF dependent mechanism

**Figure 7:**
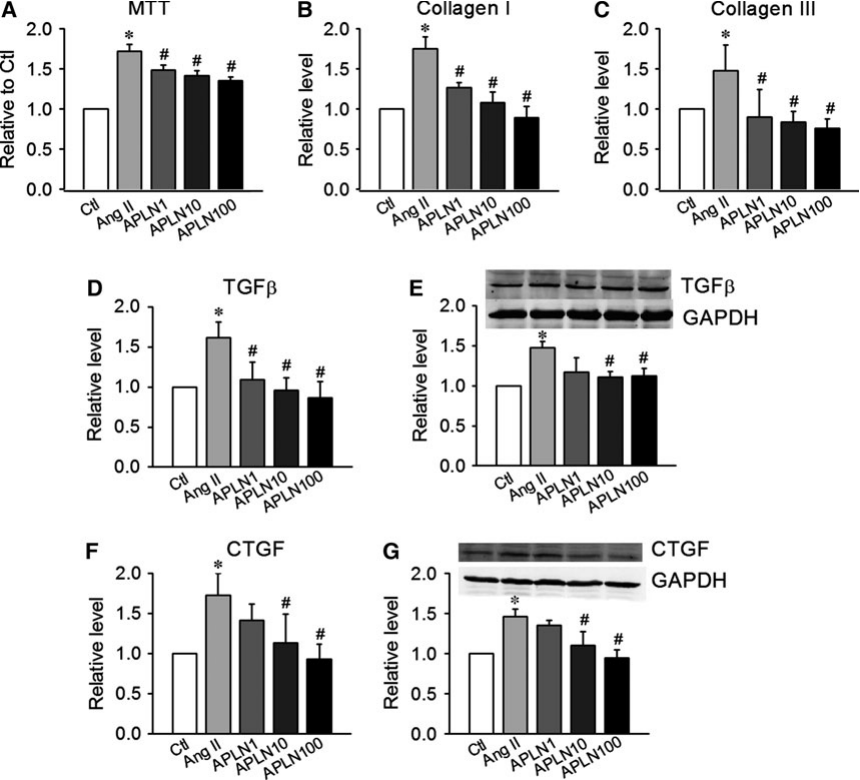
Apelin‐13 inhibits angiotensin II‐induced fibrotic responses *via* TGF‐β‐CTGF‐dependent mechanisms. (**A**) The MTT test demonstrated that Apelin‐13 (1, 10, and 100 nmol/l) inhibited the proliferation of CFs treated with Ang II (100 nmol/l) in a dose‐dependent manner. (**B** and **C**) Apelin‐13 decreased the expression levels of collagen I and collagen III in CFs treated with Ang II. (**D** and **E**) The effects of Apelin‐13 on the protein and mRNA levels of TGF‐β in CFs treated with Ang II. (**F** and **G**) The effects of Apelin‐13 on the protein and mRNA levels of CTGF in CFs treated with Ang II. Data are expressed as the means ± S.E.M.; *n* = 6. *P < 0.05 *versus* control (no treatment); #*P* < 0.05 *versus* Ang II.

Ang II promotes cardiac fibrosis, which induces collagen accumulation and contributes to cardiac remodelling. The effects of Apelin‐13 on neonatal CFs proliferation were evaluated *via* an MTT assay. Apelin‐13 (1, 10 and 100 nmol/l) significantly decreased CFs proliferation induced by Ang II (*n* = 34, *P* < 0.05) in a dose‐dependent manner (Fig. 7A). Apelin‐13 (1, 10, and 100 nmol/l) decreased the production of collagen I and III induced by Ang II (Fig. 7B and C).

In addition to blocking collagen, Apelin‐13 (1, 10, and 100 nmol/l) inhibited the angiotensin II‐induced elevation of TGF‐β and CTGF at both the mRNA and protein levels, in a dose‐dependent manner (Fig. 7D–G).

## Discussion

In this study, we found that miR‐503 was up‐regulated in mouse hearts subjected to TAC and that miR‐503 could induce the proliferation of CFs and enhance the ECM deposition, which resulted in cardiac fibrosis. Moreover, our data also strongly suggested that miR‐503 functioned as a direct epigenetic regulator of Apelin‐13.

miRNA expression has tissue specificity that determines the specific types of miRNA contributes to the biological and pathological processes of a particular organ 7. miRNA dysregulation is a common phenomenon that occurs in various diseases. miR‐503 was up‐regulated in endothelial cell from diabetic GK rats and contributes to diabetes mellitus‐induced impairment of endothelial functions and reparative angiogenesis following limb ischemia 22. ECs disfunction and, for example, endothelial to mesenchymal transition, may contribute to cardiac fibrosis. miR‐503 may be influenced by the behaviour of ECs and be involved in cardiac fibrosis. miR‐503 was down‐regulated in PAH, targeting in FGF2 and exerted antiproliferative effects in PAECs 23. Recent studies have proposed that miR‐503 is a growth suppressor in many cancer cells and overexpression of miR‐503 significantly inhibited cell proliferation, migration and invasion 24, 25, 26, 27. On the contrary, we found miR‐503 promoted myocardial fibroblasts proliferation. This result may be because of the myocardial cells belong to terminal‐differentiated cells. So the regulating mechanisms of miR‐503 were different in the two kinds of cells. In this study, we observed that the expression of miR‐503 was up‐regulated in TAC mice, as well as in proliferating CFs treated with serum or AngII. These results suggested that CFs were regulated by miR‐503. This idea was verified by the finding that the overexpression of miR‐503 promoted CFs proliferation and increased CFs collagen production.

To confirm miR‐503 function on cardiac fibrosis, we injected antagomiR‐503 in the TAC mice and evaluated their cardiac function and measure the fibrotic factors using heart tissue. Echocardiographic results showed that the antagomiR‐503 could improve the cardiac function induced by TAC. The real‐time PCR and Western Blot results showed that the antagomiR‐503 inhibited the production of collagen I and III and down‐regulated the expression of TGF and CTGF. These data *in vivo* showed that the administration of antagomiR‐503 could inhibit TAC‐induced cardiac fibrosis and supported the role of miR‐503 in heart fibrosis development. miR‐503 is a promising therapeutic target for reducing cardiac fibrosis.

Several miRNAs has been linked to regulate the development and progression of cardiac fibrosis *via* regulation of various proteins associated with fibrosis. miR‐21 levels were increased and augmented ERK‐MAP kinase activity through the inhibition of sprouty homologue 1 (Spry1) in fibroblasts of the failing heart 27. miR‐133 and miR‐30 directly down‐regulated connective tissue growth factor, a powerful inducer of ECM synthesis and regulated interstitial fibrosis. Apelin‐13 is the endogenous peptide ligand of the Apelin‐13 receptor (APJ) and has emerged as a novel endogenous counter‐regulatory mechanism of Ang II. Apelin‐13 elicits compensatory vasorelaxation under pathological conditions, including vascular endothelial dysfunction 28. Apelin‐13 protects against Ang II‐induced cardiovascular fibrosis and decreases plasminogen activator inhibitor type‐1 production 29. In this study, we explored the regulatory effects of Apelin‐13 on collagen expression and its role in mediating the fibrotic actions of miR‐503. It is well‐known that Ang II is an inducer of TGF‐β expression, so we used the neonatal CFs cultured with pre‐treatment of Ang II and then observed that the effect of Apelin‐13 on the production of collagen proteins and the expression of TGF‐β and CTGF. Pre‐treatment with Apelin‐13 inhibited the activation of CTGF and TGF‐β induced by the cotransfection of miR‐503.

To further prove the effects of miR‐503 on cardiac fibrosis by targeting APLN, in the CFs with pre‐treatment of AngII, we transfected AMO‐503 into the cells treated with siRNA‐APLN. AMO‐503 inhibited the production of collagen I and III and the expression of TGF‐β‐CTGF induced by Ang II. Cotransfection of AMO‐503 with siRNA‐APLN abolished the effects of AMO‐503 on the production of collagen I and III and the expression of TGF‐β‐CTGF. Cotransfection with siRNA‐NC did not affect the production of collagen I and III and the expression of TGF‐β and CTGF in the CFs with AMO‐503. These studies *in vitro* proved the role of miR‐503 in heart fibrosis development by APLN/TGF‐β‐CTGF pathway.

Additionally, *in silico* predictions with Targetscan demonstrated that Apelin‐13 is a target of miR‐503, a phenomenon that is conserved among species. Our results confirmed the regulation of Apelin‐13 by miR‐503 using both a luciferase reporting method and a protein expression technique. One miRNA regulates hundreds of mRNAs. Other targets of miR‐503 have been confirmed previously, including enhancers of mitogen‐activated protein kinase fibroblast growth factor 2 (basic), cyclin D2 and cyclin E1. Although our data strongly support the involvement of Apelin‐13 in miR‐503‐mediated fibrotic effects, we cannot rule out the participation of other genes in this process. The miR‐503 sequence is identical among various species, including humans and rats, implying that the effects of miR‐503 on cardiac fibrosis observed in this study may be extrapolated to human beings. Our study determined that miR‐503 modulated the expression of Apelin‐13 in the setting of fibrosis. It was reported that in pulmonary artery endothelial cells, down‐regulation miR‐503 by Apelin‐13 increased expression of FGF2 to promote endothelial cell proliferation 23. This result hinted there may be a regulating mechanism between miRNAs and protein signalling pathways. Future studies will be needed to evaluate the hypothesis.

Transforming growth factor‐β and its downstream effector, CTGF/CCN2, are fibrogenic activators. The TGF‐β‐CTGF pathway activated cells with a fibrogenetic phenotype and initiated cellular processes underlying fibrosis 30, 31. Connective tissue growth factor has also been involved in the induction of cardiac fibrosis in animal models of LV remodelling 32, 33. In our study, we observed that overexpression of miR‐503 increased the expression of TGF‐β and CTGF in CFs, an effect that was abolished by the co‐application of Apelin‐13.

In conclusion, miR‐503 decreased Apelin‐13 activity by targeting the promoter region of the Apelin‐13 gene by binding to its 3′‐UTR and up‐regulating the TGF‐β‐CTGF pathway. Down‐regulation of Apelin‐13 by miR‐503 induced production of collagen and induced cardiac fibrosis. The findings of this study highlight important clinical implications relative to miR‐503 in the setting of cardiac fibrosis.

## Conflicts of interest

The authors confirm that there are no conflicts of interest.

## Supporting information


**Figure S1** Collagen deposition induced by antagomiR‐503 in TAC mice with Masson's staining.
