# Supplementary figures and images for: MicroRNA‐503 promotes angiotensin II‐induced cardiac fibrosis by targeting Apelin‐13
Source: J Cell Mol Med. 2016 Jan 12;20(3):495–505. doi: 10.1111/jcmm.12754 (PMC4759464; doi:10.1111/jcmm.12754)

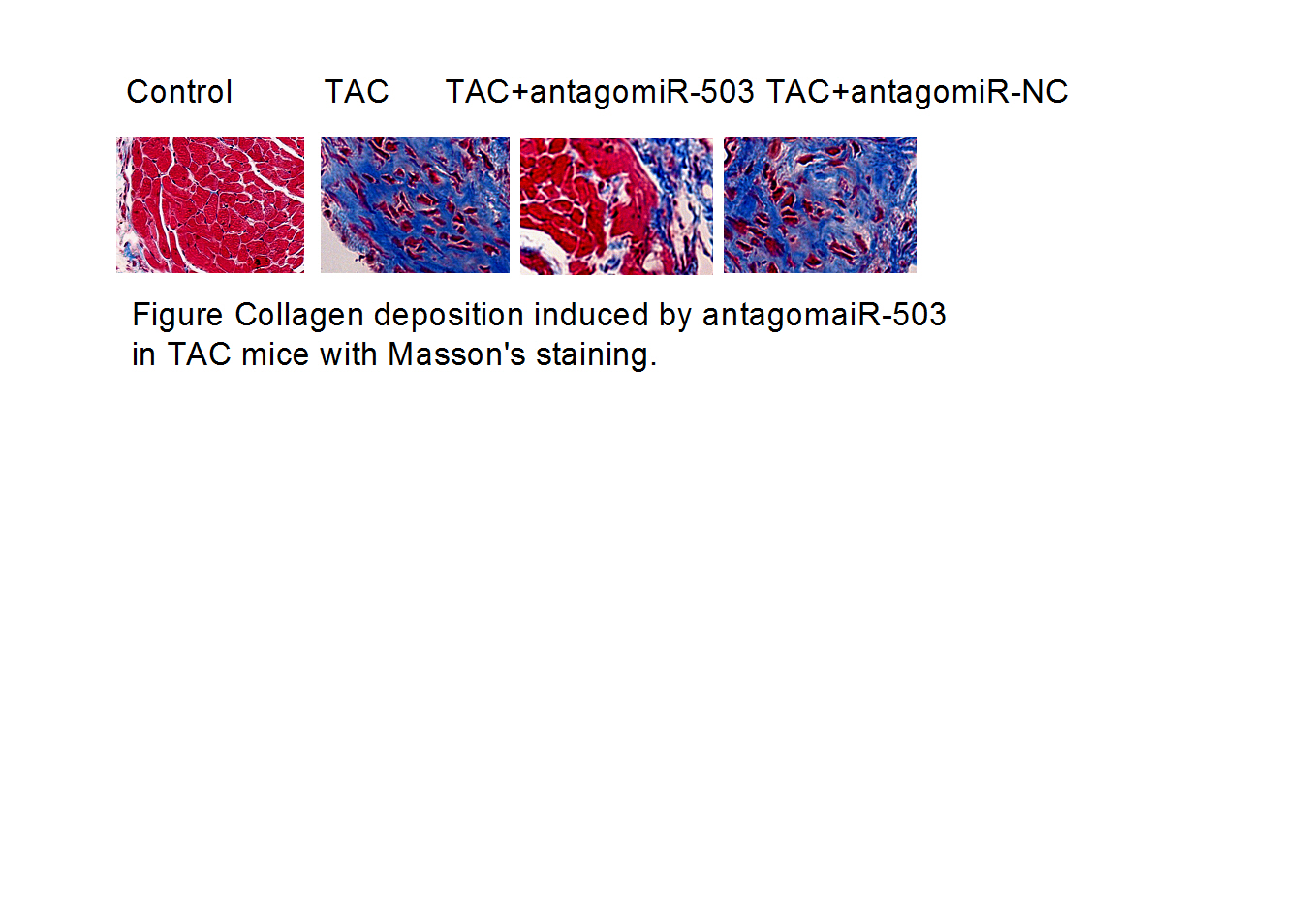

Supplement: Supplementary file 1 — Figure S1 Collagen deposition induced by antagomiR‐503 in TAC mice with Masson's staining. [file JCMM-20-495-s001.jpg]
